# End‐of‐life care over four decades in a quaternary neonatal intensive care unit

**DOI:** 10.1111/jpc.16296

**Published:** 2022-12-10

**Authors:** Alexandra Barry, Trisha Prentice, Dominic Wilkinson

**Affiliations:** ^1^ Neonatal Medicine Royal Children's Hospital Melbourne Victoria Australia; ^2^ Murdoch Children's Research Institute Melbourne Victoria Australia; ^3^ Department of Paediatrics University of Melbourne Melbourne Australia; ^4^ Oxford Uehiro Centre for Practical Ethics, Faculty of Philosophy University of Oxford Oxford United Kingdom; ^5^ Newborn Care John Radcliffe Hospital Oxford United Kingdom

## Abstract

**Aim:**

Death in the neonatal intensive care unit (NICU) commonly follows a decision to withdraw or limit life‐sustaining treatment. Advances in medicine have changed the nature of life‐sustaining interventions available and the potential prognosis for many newborn conditions. We aimed to assess changes in causes of death and end‐of‐life care over nearly four decades.

**Methods:**

A retrospective review of infants dying in the NICU was performed (2017–2020) and compared with previous audits performed in the same centre (1985–1987 and 1999–2001). Diagnoses at death were recorded for each infant as well as their apparent prognosis and any withdrawal or limitations of medical treatment.

**Results:**

In the recent epoch, there were 88 deaths out of 2084 admissions (4.2%), a reduction from the previous epochs (132/1362 (9.7%) and 111/1776 (6.2%), respectively, for epochs 1 and 2). More than 90% of infants died after withdrawal of life‐sustaining treatment, an increase from the previous two epochs (75%). There was a reduction in deaths from chromosomal abnormalities, complications related to prematurity and severe birth asphyxia.

**Conclusions:**

There continue to be changes in both the diagnoses leading to death and approaches to withdrawal of treatment in the NICU. These may reflect ongoing changes in both prenatal and post‐natal diagnostics as well as changing attitudes towards palliative care within the medical and wider community.

There have been profound changes over time in the patterns of illness, treatment and outcomes for critically ill newborn infants. Neonatal mortality has decreased, and survival is now possible for some infants born extremely prematurely or with severe malformations who would previously have died. However, some infants survive with illnesses associated with high burden of illness and treatment. Deaths in the neonatal intensive care unit (NICU) are often preceded by the difficult decision to withdraw or withhold life‐sustaining treatment and provide comfort care. Practical and ethical challenges associated with neonatal palliative care persist.^1^, ^2^, ^3^

Withdrawal of life‐sustaining treatment for some seriously ill newborns was first described in the 1970s^4^ and has manifested in different ways.^5^, ^6^, ^7^, ^8^, ^9^, ^10^, ^11^ In the early 2000s, several papers observed changes in the patterns of end‐of‐life care provision within NICUs^6^, ^7^, ^8^, ^9^, ^10^ and noted an increase in the proportion of deaths that involved decisions to limit life‐sustaining treatment. End‐of‐life care practices vary across NICUs^12^ and there remains the need for clear policies and guidance to support transparent decision‐making.^13^, ^14^, ^15^

Two retrospective reviews were conducted of infant deaths at the NICU at the Royal Children's Hospital (RCH), in the mid‐1980s and late 1990s.^16^ To ascertain how practices may have changed in the past 30 years, we replicated the previous audit to examine changing patterns over time.

## Methods

The RCH NICU in Melbourne is a 36‐bed quaternary referral centre that specialises in the care of newborn infants with complex surgical and medical conditions including genetic syndromes, congenital anomalies and complications of prematurity including severe respiratory failure.

Reviews had previously been conducted of infants who died after admission to the NICU, from July 1985 to June 1987 (24 months) and January 1999 to December 2001 (36 months). We sought to examine changes in the characteristics of dying infants by repeating the review (using identical inclusion criteria and categorisation, with the addition of further sub‐categorisations) for a third epoch from July 2017 to June 2020 (36 months). Information regarding the total number of admissions and deaths was obtained by a single author (AB) from the hospital's computerised database. Infants initially admitted to NICU who required transfer to the paediatric intensive care unit (PICU) for extracorporeal membrane oxygenation and babies transferred to Hospital in the Home were included if they remained under the neonatal bed card.

A retrospective review of electronic medical records was conducted; data were retrieved on the diagnoses of infants who died, whether treatment was withdrawn/withheld prior to death, the timing and circumstances of death and the apparent prognoses leading up to death. Prognosis was based on documented discussions on the clinical state of the patient and the expected outcomes according to the treating neonatologist, often recorded as part of family or multidisciplinary and multispecialty team meetings. Infants were then divided into groups as recorded for the previous audits (Table 1).^16^ For infants dying despite receiving maximal treatment, where death is imminent, it is common practice for life‐sustaining interventions to be withdrawn in the terminal phase (e.g. a neonate may be extubated and parents supported to hold their baby before death). In these circumstances (and consistent across epochs), the patient was classified as category I – death despite all efforts to achieve survival.

**Table 1:** Classification of infants by prognosis

Category I: Infants who died despite all efforts to achieve their survival (includes infants who were imminently dying despite maximal treatment and had resuscitation withheld)
Category II: Infants who died after withdrawal of life‐sustaining treatment
(A) Infants who would almost certainly have died even if life‐sustaining treatment had been continued
(B) Infants who would almost certainly have survived if life‐sustaining treatment had been continued
(C) Infants whose survival was not predictable and may have survived or died with ongoing life‐sustaining treatment

Within this NICU, withdrawal of life‐sustaining treatment is considered when further treatment is perceived not to be in the infant's interests and/or a decision to move to palliative care would fall in the ‘zone of parental discretion’.^17^, ^18^ Decisions around withdrawing life‐sustaining treatment are shared between parents and the treating medical team.

Infants were classified into discrete diagnostic groups as previously. Sedative and analgesic use either at, or after, withdrawal of life‐sustaining treatment and involvement of the palliative care team were also recorded. Information about the total number of admissions with certain chromosomal disorders was obtained from the unit's computerised database.

All data were anonymised and collected in accordance with institutional ethics approval.^19^

Descriptive statistics were used to analyse data. Given no hypotheses were being tested, further statistical analysis was not deemed appropriate.

## Results

There were 2084 admissions in the most recent epoch, a 17% increase from the previous period. Mortality (the proportion of admitted infants dying prior to discharge) fell over the three epochs (Table 2). In epoch 1, there was one death every 5.5 days, falling to one death every 9.9 days in epoch 2, and one every 12.4 days in epoch 3.

**Table 2:** Mortality rate for each time epoch

Total admissions	Epoch 1 (1985–1987)	Epoch 2 (1999–2001)	Epoch 3 (2017–2020)
No. admissions	1362	1776	2084
Admissions per year	681	592	695
Deaths (%) (mortality)	132 (9.7%)	111 (6.2%)	88 (4.2%)

There were changes in the diagnoses at death, with notable changes over time (Table 3).

**Table 3:** Principal diagnoses at death. Infants were assigned to a single diagnostic category based on the principal condition leading to death

Diagnoses	Epoch 1 (% of *n* = 132)	Epoch 2 (% of *n* = 111)	Epoch 3 (% of *n* = 88)
Chromosomal abnormalities	22 (16.7)	6 (5.4)	3 (3.4)
VLBW	27 (20.5)	30 (27.0)	21 (23.9)
Severe birth asphyxia	13 (9.8)	11 (9.9)	6 (6.8)
Pulmonary hypoplasia/CDH	13 (9.8)	12 (10.8)	15 (17.0)
CNS sepsis/ICH/major CNS malformation	9 (6.8)	8 (7.2)	12 (13.6)
CCHD	5 (3.8)	4 (3.6)	5 (5.7)
NTD	15 (11.4)	1 (0.9)	1 (1.1)
Miscellaneous	28 (21.2)	39 (35.1)	25 (28.4)

CCHD, complex congenital heart disease; CDH, congenital diaphragmatic hernia; CNS, central nervous system; ICH, intracranial haemorrhage; NTD, neural tube defect; VLBW, very low birthweight.

There was a reduction in the proportion of deaths related to chromosomal abnormalities over time. In the last epoch, all three deaths in this category were infants with antenatally diagnosed trisomy 21 complicated by complex medical problems unresponsive to intensive treatment. This contrasts to epochs 1 and 2 (trisomy 18: 7 and 1 deaths, respectively; trisomy 13: 4 and 3 deaths). Simultaneously, there was a reduction in the number of patients admitted with trisomies. In epoch 3, there were 12 admissions with trisomy 21 (half of whom were post‐natal diagnoses) compared with 19 and 25, respectively for epochs 1 and 2. There was only one admission with trisomy 18 (postnatally diagnosed) in epoch 3; the baby was subsequently transferred to a regional hospital for ongoing palliative care. A sustained reduction in the number of deaths from neural tube defects was also noted (15 in epoch 1, 1 each in epochs 2 and 3) that coincided with a decrease in the number of admissions associated with this condition (41, 7 and 1 in epochs 1, 2 and 3). Additionally, a decline in deaths associated with severe birth asphyxia was noted, in line with a reduction in admissions with this condition.

An increase was seen in the number and proportion of deaths related to pulmonary hypoplasia, congenital diaphragmatic hernia, and intracranial structural abnormalities, in line with an increase in respective admissions. An initial increase was noted from epochs 1 to 2 in the proportion of deaths related to complications of extreme prematurity, followed by a subsequent decline for epoch 3. About 12 out of 21 of the VLBW deaths in epoch 3 were attributed to necrotising enterocolitis (NEC).

Further diagnostic breakdown among the miscellaneous diagnoses was available for epochs 2 and 3 but not epoch 1 (Table 4).

**Table 4:** Miscellaneous diagnoses breakdown for epochs 2 and 3

Miscellaneous breakdown	Epoch 2 (% of *n* = 39)	Epoch 3 (% of *n* = 25)
Syndrome, e.g., CHARGE, VACTERL	10 (25.6)	8 (32)
Metabolic	4 (10.3)	3 (12)
Neuromuscular	1 (2.6)	2 (8)
Isolated genetic, e.g., EB	7 (17.9)	8 (32)
Sepsis (non‐CNS)	8 (20.5)	3 (12)
Respiratory, e.g., mec asp	8 (20.5)	0
NEC	1 (2.6)	0
Oncology	0	1 (4)

CNS, central nervous system; EB, epidermolysis bullosa; mec asp, meconium aspiration; NEC, necrotising enterocolitis.

**Fig. 1:**
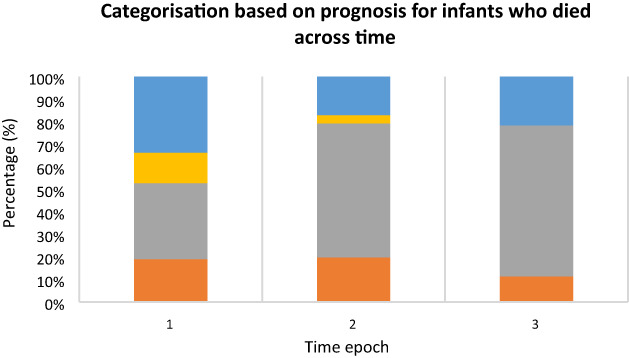
Percentage of infants who died in each prognosis category across time. (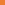), Category I; (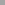), IIa; (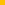), IIb and (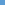), IIc

In the most recent epoch, there was a further reduction in the proportion of infants who died despite maximal treatment and 91 % of infants died following limitation of life‐sustaining treatment (Table 5, Fig. 1).

**Table 5:** Categorisation based on prognosis for infants who died in each epoch

Category	Epoch 1 (% of n = 132)	Epoch 2 (% of *n* = 111)	Epoch 3 (% of *n* = 88)
Category I	31 (23.5)	22 (19.8)	8 (9.1)
Category II	101 (76.5)	89 (80.2)	80 (90.9)
IIa	42 (31.8)	66 (59.5)	61 (69.3)
IIb	17 (12.9)	4 (3.6)	–
IIc	42 (31.8)	19 (17.1)	19 (21.6)

There was an increase in the proportion who had treatment withdrawn in the expectation that they would almost certainly die even if treatment were continued. There were no infants in the third epoch for whom medium/long‐term survival was expected if treatment were continued (compared to 13% of deaths in the first epoch).

Infants in the third epoch were older at the time of palliation (Table 6). About 85% of deaths occurred in the NICU, while the remainder were split between PICU (10%), another ward or other hospital (2%) and home (3%).

**Table 6:** Age at time of withdrawal of life‐sustaining treatment

	Epoch 1	Epoch 2	Epoch 3
Age at time of withdrawal of life‐sustaining treatments – mean in days (range)	10.4 (1–121)	24.3 (1–226)	39.1 (1–198)

In the most recent era, 86% of infants who had limitations of treatment had mechanical ventilation withdrawn (an increase compared with earlier epochs) (Table 7). There was a substantial reduction in the proportion of deaths following withdrawal of enteral nutrition. Almost all infants (97.7%) in the current epoch were receiving analgesics and/or sedatives at the time of treatment limitation, with the remainder being unexpected deaths. This was an increase from 66% and 85%, respectively, for epochs 1 and 2.

**Table 7:** Principal modality of treatment withdrawn (denominator: all category II infants)

Modality withdrawn	Epoch 1	Epoch 2	Epoch 3
Ventilation	40 (40%)	69 (78%)	69 (86%)
Supplemental O_2_	8 (8%)	0	3 (4%)
Artificial nutrition	48 (48%)	12 (14%)	8 (10%)
Cardiopulmonary resuscitation withheld	0	3 (3%)	0
Other	5 (5%)	5 (6%)	0

The hospital palliative care team was involved in 17.5% of patients in the current epoch (not recorded in previous audits). Post‐mortem examination occurred in 30% of deaths in the most recent epoch, similar to the second, but lower than in the first epoch (58%).

In all cases, treatment was withdrawn with the agreement of parents; however, in one quarter of recent cases, parents initially wished for treatment to continue (data not available for earlier epochs). For patients who were imminently dying despite maximum treatment, palliative extubation was declined in 3/8 (75%). Among the category 2 infants, 19/80 (23.8%) parents initially declined and then subsequently agreed to withdrawal of life‐sustaining‐therapies.

## Discussion

We have documented changes in the causes and circumstances of death over a 35‐year period in a single specialised NICU. There has been an increase in the number of NICU admissions over time but a decrease in the mortality rate. More than 90% of deaths in the most recent epoch occurred following withdrawal of life‐sustaining treatment, characterised mainly by withdrawal of mechanical ventilation. Sustained disagreement between clinicians and families about end‐of‐life decisions was uncommon.

The proportion of deaths following treatment withdrawal is higher than in other studies in the past two decades in Europe and the United States (38–72%),^6^, ^7^, ^9^, ^10^, ^20^, ^21^ including perinatal and surgical centres. An increase in withdrawal of active treatment was noted over time in all these papers. A more recent study looking at end‐of‐life care in two NICUs in Canada found that 81% of infants died following withdrawal of ventilatory support, similar to the most recent epoch of our study.^12^ The relatively high proportion of deaths following a decision to withdraw treatment may reflect the prevailing values of clinicians and families as well as the complex congenital and surgical background of many of our patients. Of note, the perception that most infants would have died even if life‐sustaining interventions were continued suggests decisions predominately centred around illness severity rather than quality of life considerations.

One end‐of‐life practice that was relatively frequent in neonatal intensive care in the 1980s is now uncommon. In the first epoch, 36% of deaths (48% of treatment withdrawal) were associated with withholding of artificial nutrition, while in the most recent period, less than 10% of deaths fell into this category. This may reflect the relative illness severity of this latest cohort in addition to changes in attitudes and practice. Though withdrawal of artificial nutrition and hydration remains (in certain circumstances) an ethically permissible option within palliative care practice, there has simultaneously been a shift towards providing long‐term TPN beyond the neonatal period for severe gastrointestinal pathologies once deemed life‐limiting (e.g. extremely short gut) unless there are complicating factors such as difficult intravenous access, liver disease or infection.^22^, ^23^

Causes of death in the NICU also changed with time. The persistent fall in the proportion of deaths due to chromosomal abnormalities is likely to be related at least in part to antenatal detection and subsequent termination of pregnancy. A study of perinatal deaths in Western Australia (1986–2010) demonstrated a significant rise in the number of terminations, which was associated with shifting stillbirth and neonatal death rates.^24^ An earlier study in Victoria (1989–2000) found a fall in overall perinatal mortality related to the termination of foetuses with severe birth defects.^25^ There have also been previous reports of reductions in mortality for infants with trisomy 21.^26^ Antenatal diagnosis and management in perinatal centres may also have reduced admissions with severe trisomies.

While many severe chromosomal abnormalities are diagnosed antenatally by invasive or non‐invasive means, there have also been changes related to advances in genetic testing. Whole‐exome sequencing (WES) and genome sequencing are increasingly used for infants with suspected monogenic disease, providing earlier diagnostic certainty when compared with standard investigations.^27^ Early confirmation of inherited life‐limiting conditions has implications for genetic counselling and family planning.^28^ While this genetic sequencing rapidly evolved during epoch 3, rapid genomic sequencing (RGS, <72 h turnaround) was not yet available in many instances where WES was performed, and end‐of‐life decisions had already been made on clinical grounds.^29^ The effect of genomic sequencing on end‐of‐life decision‐making is thus not yet apparent within this cohort.

The observed fall in the proportion of severe birth asphyxia deaths could relate to changes in treatment. A large multi‐centre Spanish study observed a slight decline in mortality from hypoxic‐ischaemic encephalopathy mortality from 2011 to 2019 associated with the use of therapeutic hypothermia.^30^ However, the observed change in our study may be more linked to changes in admission patterns. Infants with HIE in Victoria are usually managed in perinatal centres, with our centre increasingly focused on specialised complex surgical and medical conditions. Complications from extreme prematurity continue to represent a prominent cause of death, as seen in other NICUs.^12^

Use of comfort medications in the form of analgesia or sedatives was comparable to the end‐of‐life care literature.^12^, ^31^, ^32^ The increased use of either analgesics or sedatives across the 3 epochs may reflect changes in neonatal pain management and an increased awareness of the need for pain relief with common interventions in the NICU.

In the most recent cohort, we were able to observe the involvement of the specialised palliative care team. Despite recognition of the importance of palliative care practices in NICUs,^15^ only a small proportion of deaths were accompanied by a palliative care referral. This reflects the local model of care: neonatal clinicians are upskilled in the provision of end‐of‐life care in accordance with international recommendations for perinatal palliative care.^15^ Specialty palliative care involvement is only sought if the dying process is anticipated to be prolonged, palliative care is to occur in the community or a specialised symptom management plan is required. Patients discharged to palliative care within the community may not have necessarily been captured by this study as the patients were no longer under the neonatal bed card.

As this study is based in a single centre with a highly specialised referral base, the findings may not generalise to other neonatal units, or to other parts of the world. We deliberately sought to replicate the methods of the earlier study (initially commenced in 1985) to enable comparison. However, there may be discrepancies in the data collection or interpretation related to different people collecting the data in each epoch. Prognosis was based on documented conversations and may be interpreted differently by a different clinician. Furthermore, in the current era, an alternative categorisation of diagnosis, prognosis or end‐of‐life decisions might be preferred. For example, physiological status before death as described by Verhagen *et al*.^33^ As noted above, changes in cause of mortality may reflect either changes in referral patterns or to treatment decisions, and it is difficult to disentangle these. We did not have access to the outcome of infants transferred or discharged. It is possible that the study missed deaths in infants transferred to other units or discharged with palliative care.

## Conclusion

This study provides an important update on end‐of‐life care in a NICU, with insights into how practices have changed over time. While the overall mortality for babies admitted to the NICU has decreased, death remains a relatively common event. An increasing number of these deaths follow a decision to withdraw or limit life‐sustaining treatment, highlighting the importance of a clear, coordinated approach to palliative care in neonates. Initial disagreement around end‐of‐life decisions was ultimately resolved in all cases.
